# Bipolar Radiofrequency Facet Ablation of the Lumbar Facet Capsule: An Adjunct to Conventional Radiofrequency Ablation for Pain Management

**DOI:** 10.7759/cureus.1635

**Published:** 2017-09-01

**Authors:** Robert E Jacobson, Ovidiu Palea, Michelle Granville

**Affiliations:** 1 Miami Neurosurgical Center, University of Miami Hospital; 2 Anesthesiology and Pain Management, Provita Hospital

**Keywords:** radiofrequency facet ablation, facet cysts, pain control, lumbar facet capsule thermocoagulation, lumbar facet cysts, lumbar facet osteoarthritis, facet joint pain, facet joint degeneration

## Abstract

Radiofrequency facet ablation (RFA) has been performed using the same technique for over 50 years. Except for variations in electrode size, tip shape, and change in radiofrequency (RF) stimulation parameters, using standard, pulsed, and cooled RF wavelengths, the target points have remained absolutely unchanged from the original work describing RFA for lumbar pain control. Degenerative changes in the facet joint and capsule are the primary location for the majority of lumbar segmental pathology and pain. Multiple studies show that the degenerated facet joint is richly innervated as a result of the inflammatory overgrowth of the synovium. The primary provocative clinical test to justify an RFA is to perform an injection with local anesthetic into the facet joint and the posterior capsule and confirm pain relief. However, after a positive response, the radiofrequency lesion is made not to the facet joint but to the more proximal fine nerve branches that innervate the joint. The accepted target points for the recurrent sensory branch ignore the characteristic rich innervation of the pathologic lumbar facet capsule and assume that lesioning of these recurrent branches is sufficient to denervate the painful pathologic facet joint. This report describes the additional targets and technical steps for further coagulation points along the posterior capsule of the lumbar facet joint and the physiologic studies of the advantage of the bipolar radiofrequency current in this location. Bipolar RF to the facet capsule is a simple, extra step that easily creates a large thermo-coagulated lesion in this capsule region of the pathologic facet joint. Early studies demonstrate bipolar RF to the facet capsule can provide long-term pain relief when used alone for specific localized facet joint pain, to coagulate lumbar facet cysts to prevent recurrence, and to get more extensive pain control by combining it with traditional lumbar RFA, especially when RFA is repeated.

## Introduction

The facet joint has been identified as a cause of lumbar pain as long as the spine has been studied both anatomically and clinically. In fact, before the lumbar disc was described by Mixter and Barr in 1933 as a cause of root compression, primary attention was directed towards the facet joint, both as a cause of lumbar and paraspinal pain and as a cause of nerve root compression in the neural foramen. Von Luschka in anatomic dissections in the 1860s described both the facet joints and small recurrent branches of the dorsal sensory nerve root originating posteriorly to form a diffuse plexus over the lumbar facet capsule [1]. The facet joints have been clearly recognized as a critical mechanical structure in segmental spinal motion [2-3]. These small synovial joints are vulnerable to significant inflammatory and degenerative changes [4]. Clinical observations using hypertonic saline and findings of pain relief after making small incisions around the facet joint were later adapted to use radiofrequency ablation (RFA) with heat as a simple percutaneous procedure targeting the dorsal recurrent sensory branch for relief from back pain [5-6]. These targets for RFA were based on von Luschka's anatomic work [1,7]. However, anatomic dissections in pathologic spinal segments have shown that these more proximal dorsal innervations of the recurrent sensory nerve have significant variability and can be affected by bone osteophytes [7-8]. More detailed anatomic and physiologic studies of these dorsal 'sensory' nerves actually reveal a more complicated pattern of innervation with wider distribution around the pathologic facet capsule and hypertrophied synovium of the facet joint [9]. Despite these anatomic and pathologic findings indicating a very rich innervation of the degenerated facet joint, the facet capsule has been ignored as either a primary or a supplementary target for radiofrequency modulation for pain control [2,5]. This is the rationale for adding the lumbar facet capsule as a target for radiofrequency ablation.

## Technical report

Step one: Radiofrequency ablation of the recurrent dorsal sensory branches (RFA)

The patient is positioned prone on abdominal rolls. The anesthesiologist can give light IV sedation, as required. Using anteroposterior (AP) or slightly oblique fluoroscopic guidance, the posterior facet joint is radiologically identified. Using the standard RF target point at the medial base of the transverse process as it joins the base of the superior facet after standard sterile preparation and draping, the skin entry points for placing the electrodes are anesthetized with one percent lidocaine. At each target point for RFA, a 10 mm regular tip 150 mm length electrode or a Venom electrode (Stryker, Kalamazoo, MI, USA) is positioned. Four to six electrodes are positioned covering at least two vertebrae and used with the MultiGen Radiofrequency Generator (Stryker, Kalamazoo, MI, USA). Once bone contact is made sensory, motor testing up to three mV is performed before any lesioning to ensure the safe positioning of the electrodes. Lesioning is performed at the targeted vertebral level and, at the minimum, in the segment above and below using four to eight electrodes. A standard RFA lesion at 80 ^o^C for 90 seconds is made using r electrodes at a time. After performing radiofrequency lesioning, 1 cc of 0.5% bupivacaine is injected down the RF electrode cannula before it is withdrawn.

Step two: Bipolar radiofrequency lesioning of the facet capsule and facet joint

**Figure 1:**
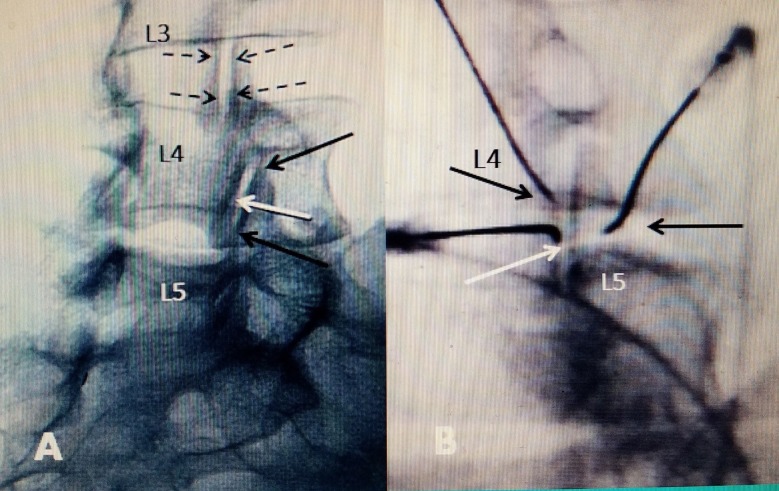
Positioning of bipolar RF electrodes Electrodes positioned within 10 mm of each other for bipolar effect A: At L3-4, dotted black arrows represent the positioning of electrodes on either side of the facet joint capsule. The capsule covers the posterior joint and radiofrequency (RF) current heats across the posterior capsule. At the L4-5 facet joint, the two bipolar electrodes represented by solid black arrows are positioned lengthwise along the superior and the inferior part of the joint space, creating a bipolar RF effect along the length of joint. One electrode is on the edge of the inferior facet L4 and the other on the superior facet of L5 along the L4-5 joint space. The solid white arrow represents an intrafacet electrode positioning within the joint space. B: Oblique radiograph during the actual procedure covering the L4-5 fact joint. Black arrows on two RF electrodes on either side of the joint space are the two bipolar electrodes across the L4-5 joint space. The white arrow is an RF electrode with a curved tip positioned within the joint space.

After RFA, the RF electrode is partially retracted along the lateral wall of the superior facet and then angled slightly medially toward the radiologic line of the facet joint space identified under fluoroscopy. This repositions the electrode to the mid-dorsal surface of the posterior facet capsule. Depending on the size of the posterior facet joint, as seen under fluoroscopy and confirmed on review of the patient's axial computed tomography (CT) or magnetic resonance imaging (MRI) scan, two or four additional electrodes are positioned on either side of each facet joint. Either a standard 10 mm exposed tip or Venom electrodes can be used. If the Venom electrode is used, when deployed, there is a wider five mm exposed inverted ‘V’ tip providing a larger area of thermocoagulation. Regardless of the electrode used, since there is no nerve root or significant sensory or motor branch near the posterior capsule, no further sensory or motor testing is needed. After proper positioning of the electrodes arrayed along the target facet joint capsule, lesions are made at 80 ^o^C for 90 seconds, similar to RFA. The second approach is to position two to four electrodes parallel to each other along the length of the facet capsule and directly into the posterior joint if it is wide enough in a sagittal plane. Radiofrequency lesions are made for 80 ^o^C for 90 seconds. This approach with two adjacent or parallel electrodes positioned between 10 and 12 mm apart creates a bipolar lesion with broader denervation of the fine nerve endings within the abnormal facet capsule (Figure 1) [10].

## Discussion

**Figure 2:**
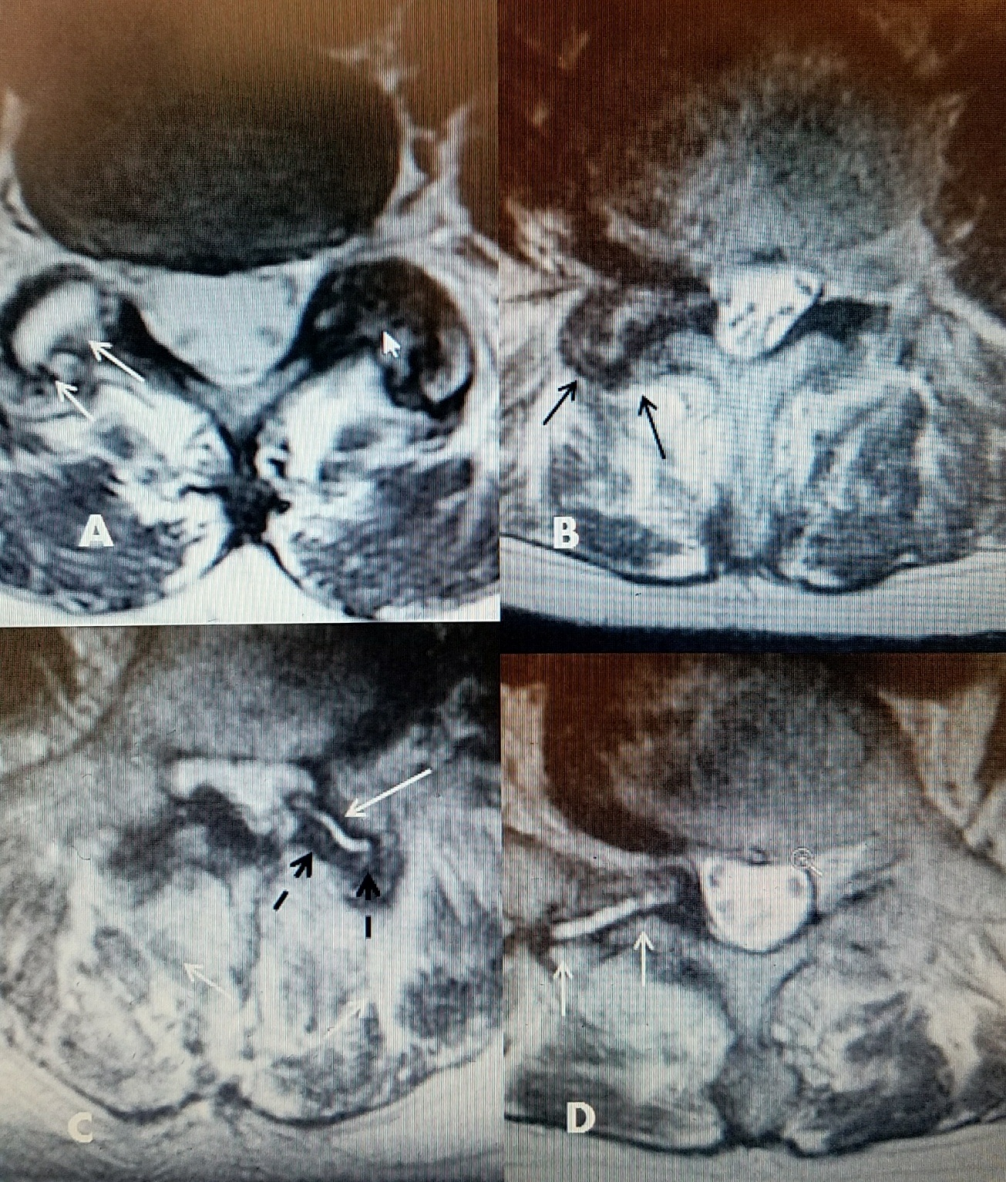
Axial MRI images of the lumbar facet joints showing fluid, osteoarthritis, and facet capsule enlargement A: L5-S1 bilateral abnormal facets. There is hypodense fluid signal in one facet joint (two white arrows) with an enlarged joint space. The hypertrophied facet is on the opposite side with reactive bone overgrowth (small solid white arrow). B: L4-5 marked degenerative facet hypertrophy (solid black arrows), causing severe foramena and lateral recess stenosis. C: L4-5 unilateral facet fluid and enlargement of capsule into the ventral spinal canal, causing lateral recess stenosis (white arrow). Hypertrophied posterior capsule and bone (dashed black arrows). There is thickening of the ligamentum flavum posteriorly on the opposite side. D: Unilateral enlarged L5-S1 facet with fluid (white hypodense signal on T2 magnetic resonance imaging (MRI)), causing foramena and lateral recess stenosis (solid white arrows).

The physics and physiologic effects of radiofrequency current for thermocoagulation have been studied extensively, resulting in technical variations in making finer electrodes and different electrode tips and varying the stimulation parameters in an effort to improve the effects of radiofrequency lesioning. In other areas of the body, radiofrequency has been developed extensively for different targets, such as cardiac lesioning, ablation of soft tissue tumors in the liver and kidney, and in various orthopedic, vertebral, and epidural tumors [10]. Since the original work describing the use of RFA for pain control in the 1970s, radiologic diagnosis of spinal disease has become much more precise with the use of computerized tomography (CT) and magnetic resonance imaging (MRI). Repeated biomechanical stress and chronic segmental disc degeneration cause inflammatory soft tissue and bone changes around the facet joint [3-4]. As the degenerated facet joint enlarges, there develops a rich periarticular innervation associated with swelling of the synovium of the facet capsule. Pathologic studies demonstrate a much more diffuse distribution of fine nerve endings than described by von Luschka in the posterior-medial joint capsule as well as the enlarged synovial folds of the facet capsule with both sensory and autonomic nerve fibers [9]. These submillimeter para-vascular myelinated nerve fibers course through the synovial folds, not in association with blood vessels, suggesting that these nerves are nociceptive and may have clinical significance in spinal and especially facet pain [11]. Interestingly, there are no nerve endings within the ligamentum flavum even though it is directly attached to the medial and ventral edge of the facet joint. As a result, enlargement of the ligament flavum does not directly cause pain but rather symptoms from nerve root compression in the lateral recess or from spinal stenosis [11]. There is also a rich micro-innervation that is found deep within the joint itself, connected to the synovium capsule [4,11-12]. Angiogenesis with nerve in-growth has been documented with both degenerative inflammation and arthritis of small and large synovial joints and contributes to an increasing nerve growth and joint pain [13].The normal facet joint is a small synovial joint, at most holding 0.75 to 1.5 cc of fluid. A frequent characteristic of facet degeneration is excess fluid within the joint space, often associated with various size cyst formations [3-4]. Studies of osteoarthritis of both large and small synovial joints show an elevation of pain producing peptides and cytokines in this joint fluid [12]. The facet joint develops similar subchondral and capsular pathologic changes that are seen in large joints such as the hip and knee. However, since the facet joint is so small compared to a knee, hip, or shoulder, these changes to the subchondral bone and the attachments of the joint capsule make up a proportionately larger part of the pathologic joint surface. It is important to note that facet osteophytes (bone spurs) have been shown only to occur on the periphery of synovial joints leading to irregular enlargement of the joint surface, which are the targets for additional radiofrequency lesioning (Figure 2).

Injecting the facet capsule with a local anesthetic to evaluate if there is positive pain relief is the provocative test for evaluating joint pain, and determining if the patient is subsequently a potential candidate for RFA is targeting the facet joint itself [14]. As previously described, lumbar degenerative osteoarthritis is associated with enlargement of the facet joint and capsule, which is the main source of localized back and facet pain. Many large studies of RFA of the dorsal recurrent sensory branch have shown mixed results and often the need to repeat the RFA within 8 to 10 months [2,15-16]. One possible reason for this mixed outcome lies in the nature of the pathologic facet joint, which is enlarged and develops a fine web of abnormal nerve in-growths in the synovium of the facet capsule and the hypertrophied bone [11-13]. These fine nerve endings are probably not directly addressed by the accepted targets for RFA. These original target points do not take into consideration the pathology and anatomy of the facet joint described in this report and is easily recognized today on axial CT and MRI scans [2-3,6-7]. The peripheral parts of the hypertrophic facet capsule may require more direct denervation than is provided by targeting the recurrent nerve branches. In targeting the facet capsule for radiofrequency thermocoagulation to denervate the multiple fine nerve endings, there is not one specific point to apply the radiofrequency heat as compared to the traditional lesioning of the recurrent branch [6-8]. It is clear, looking at anatomic and pathologic studies, that multiple sites along the length of the posterior capsule, including both the most medial and posterior edges where bone spurs form, must be treated with radiofrequency to cover the wider web of abnormal nerve endings that develop with facet arthropathy [12-15]. It has been shown that by approximating two electrodes in parallel, a consistently larger lesion is made. These bipolar lesions effectively raise the temperature and coagulate all intervening soft tissue [10]. In the technique described in this report, by simply repositioning the original regular RFA needle from the base of the superior facet and transverse process to the posterior facet joint capsule and adding other electrodes to create a bipolar thermal effect, it is easy to perform almost complete radiofrequency lesioning of the facet capsule and joint at the same sitting after the RFA is performed. This larger lesion is able to cover the entire facet capsule on both sides of the facet joint and can include the hypertrophied joint edges [10,17-18]. Radiofrequency thermocoagulation can also be applied within the joint, especially if there is enlargement of the joint space with intra-articular fluid. This joint fluid in one or both facets is often seen on axial MRI scans or in cases with development of facet cysts. Radiofrequency thermocoagulation can be used after cyst drainage to reduce the incidence of cyst recurrence [19]. Pulsed radiofrequency has been used directly to the synovium and within large and small joints elsewhere in the body for pain control [20]. Pulsed radiofrequency has not been reported directly in the facet joint, but it can be used as described in this report both to the facet capsule and within the joint space when it is wide enough to place an electrode. By applying multiple radiofrequency electrodes to create sufficient heat lesions along the abnormal joint capsule alone or combined with regular RFA targets, bipolar radiofrequency targeting of the lumbar facet capsule is a reasonable adjunct to standard RFA. Results over time will show if this an improvement and especially an advantage in patients that may need repeat RFA.

## Conclusions

Since the original development of radiofrequency facet ablation for the thermocoagulation of the lumbar dorsal medial recurrent sensory branch for back and facet pain, there has been no change in the original lesioning targets at the base of the transverse process and superior facet. There have been many refinements in radiofrequency electrode design and stimulation parameters without significant change in the overall results from RFA. This article reviews the many changes in the understanding of the anatomy and biomechanics of the lumbar facet joint and the pathologic changes seen with CT and MRI and how this may affect the approach to the use of radiofrequency thermocoagulation in treating facet joint pain. Recognition of the abnormal nerve in-growth and inflammatory angiogenesis that occur with the degenerative changes around inflamed and arthritic synovial joints makes these fine nerve endings additional targets for pain control. Adding the facet capsule is a simple additional target for both primary lumbar pain management and especially for cases of recurrent pain after successful RFA. By using parallel placement along either the facet posterior capsule or the edges of the facet joint, the two electrodes create a bipolar lesion that is directed at the abnormal nerve endings in the facet joint capsule. These additional lesions can be performed along with regular RFA of the recurrent branch or separately. This is a simple step that has significant advantages, no added side effects, and, in preliminary cases, shows better initial and follow-up results.
